# Mexican dataset of digital mammograms (MEXBreast) with suspicious clusters of microcalcifications

**DOI:** 10.1016/j.dib.2025.111587

**Published:** 2025-04-28

**Authors:** Ricardo Salvador Luna Lozoya, Karina Núnez Barragán, Humberto de Jesús Ochoa Domínguez, Juan Humberto Sossa Azuela, Vianey Guadalupe Cruz Sánchez, Osslan Osiris Vergara Villegas

**Affiliations:** a Universidad Autónoma de Ciudad Juárez, Instituto de Ingeniería y Tecnología, Ciudad Juárez, Chihuahua, Mexico; b Radiodiagnóstico e Imagen de Chihuahua Clinic, Chihuahua, Chihuahua, Mexico; c Instituto Politécnico Nacional, Centro de Investigación en Computación, Mexico City, Mexico

**Keywords:** Breast cancer, BI-RADS 4, Imaging resolution, Deep learning

## Abstract

Breast cancer is one of the most prevalent cancers affecting women worldwide. Early detection and treatment are crucial in significantly reducing mortality rates Microcalcifications (MCs) are of particular importance among the various breast lesions. These tiny calcium deposits within breast tissue are present in approximately 30% of malignant tumors and can serve as critical indirect indicators of early-stage breast cancer. Three or more MCs within an area of 1 cm² are considered a Microcalcification Cluster (MCC) and assigned a BI-RADS category 4, indicating a suspicion of malignancy. Mammography is the most used technique for breast cancer detection. Approximately one in two mammograms showing MCCs is confirmed as cancerous through biopsy. MCCs are challenging to detect, even for experienced radiologists, underscoring the need for computer-aided detection tools such as Convolutional Neural Networks (CNNs). CNNs require large amounts of domain-specific data with consistent resolutions for effective training. However, most publicly available mammogram datasets either lack resolution information or are compiled from heterogeneous sources. Additionally, MCCs are often either unlabeled or sparsely represented in these datasets, limiting their utility for training CNNs. In this dataset, we present the MEXBreast, an annotated MCCs Mexican digital mammogram database, containing images from resolutions of 50, 70, and 100 microns. MEXBreast aims to support the training, validation, and testing of deep learning CNNs.

Specifications Table

**Table:** 

Subject	Computer Sciences
Specific subject area	Deep learning.
Type of data	Table, Image.
Data collection	The mammograms were sourced from the Radiodiagnóstico e Imagen de Chihuahua clinic in Chihuahua, Chih., Mexico, via their Picture Archiving and Communication System (PACS). Two radiologists from the clinic manually labeled the mammograms for Microcalcification Clusters (MCCs). Subsequently, the images were converted from the DICOM format to PNG format.
Data source location	Country: Mexico.
Data accessibility	Repository name: Mendeley Data Data identification number: 10.17632/ym4mm9xvhm.2 Direct URL to data: https://doi.org/10.17632/ym4mm9xvhm.2
Related research article	R. Luna, H. Ochoa, H. Sossa, V. Cruz, O. Vergara, Residual shallow convolutional neural network to classify microcalcifications clusters in digital mammograms, Biomedical Signal Processing and Control. 102 (2025) 107209. https://doi.org/10.1016/j.bspc.2024.107209 [1]

## 1 Value of the Data

• The dataset is highly valuable as it contains a sufficient number of direct PNG images to train, validate, and test Convolutional Neural Networks (CNNs) at three distinct resolutions, eliminating the need for data augmentation for the detection or classification of Microcalcification Clusters (MCCs). Additionally, it is the only publicly available Mexican database with labeled MCCs. Furthermore, the accompanying table provides detailed information about each mammogram, including scanner specifications, image dimensions, resolution, laterality, view, BI-RADS code, lesion type, and the dimensions and coordinates of MCCs within the mammogram.
• As a public database, it is accessible to anyone interested in leveraging deep learning models to detect, classify, generate, or analyze MCCs. The mammograms are provided in PNG format, enabling immediate use without requiring third-party software to convert from DICOM to PNG, a common prerequisite when working with CNNs. Furthermore, all relevant information for each mammogram is conveniently consolidated in a single CSV file, eliminating the need to explore each image to extract such data manually.
• By including the most commonly used resolutions, researchers can validate their models trained on other datasets using MEXBreast, enhancing the robustness and generalizability of their research findings.
• Each mammogram was interpreted and labeled by two expert radiologists specializing in breast imaging, ensuring a curated database with high reliability and accuracy.
• Each mammogram has been optimized by removing embedded labels, such as patient information, scanner specifications, and additional annotations overlaid on the image. This enhancement improves image clarity and ensures that algorithms examine clinically relevant areas, potentially reducing noise and increasing the accuracy of automated assessments.
• Each mammogram has been standardized to grayscale, with pixel intensity values ranging from 0 to 255, ensuring uniformity for consistent analysis and processing.

## 2 Background

As researchers, one of the most challenging tasks in detecting MCCs is finding publicly available databases of digital mammograms Among these, it is crucial to ensure that the databases include mammogram resolution specifications. This requirement arises because training a CNN model demands consistency in the input domain. Unfortunately, this resolution parameter is rarely available in public databases unless the images are provided in a medical format like DICOM. The DICOM format includes metadata tags, one of which specifies a resolution, but this presents another challenge: most of the available databases are compiled from mixed sources, requiring a manual search to determine the resolution of each image individually. In addition to these challenges, we require databases where MCCs are clearly identified or annotated, which is rarely the case. To address these limitations, we developed the MEXBreast database, a curated resource specifically designed to be ready for use in automated tasks such as classification and detection. MEXBreast not only provides the identification of resolution across all images but also ensures the presence of MCC annotations, making it an invaluable tool for researchers working on advanced AI applications in mammography.

## 3 Data description

Here is the information included in this dataset:

MARKED MAMMOGRAMS folder: This folder contains 620 mammograms in PNG format, with MCCs highlighted using bounding boxes. It serves as a visual reference to easily locate these lesions.

UNMARKED MAMMOGRAMS folder: This folder contains the same 620 mammograms in PNG format as the MARKED folder but without any markings or annotations. These mammograms are suitable for conducting experiments.

DATA File: This comma-separated file provides detailed information about each of the 620 mammograms. Table 1 provides the description of each field or column in the file.

**Table 1:** Attributes and descriptions of the DATA File.

Column Title	Description
Image Name	Name of the mammogram image file.
Scanner Manufacturer	Manufacturer of the scanner used to capture the mammogram.
Scanner Model	Model of the scanner used to capture the mammogram.
Image Height (px)	Height of the mammogram image in pixels.
Image Width (px)	Width of the mammogram image in pixels.
Pixel Resolution (mm)	Pixel resolution of the image in millimeters.
Patient Age	Age of the patient at the time of the mammogram.
Laterality	Laterality of the breast imaged (e.g., left or right).
View	Type of view for the mammogram (e.g., CC or MLO)*.
BI-RADS Category	BI-RADS category assigned to the mammogram.
Lesion Type (ROI)	Type of lesion in the ROI^⁎⁎^.
ROI Height (px)	Height of the ROI in pixels.
ROI Width (px)	Width of the ROI in pixels.
ROI Y1 (px)	Y-coordinate of the top boundary of the ROI in pixels.
ROI Y2 (px)	Y-coordinate of the bottom boundary of the ROI in pixels.
ROI X1 (px)	X-coordinate of the left boundary of the ROI in pixels.
ROI X2 (px)	X-coordinate of the right boundary of the ROI in pixels.

*CC: Craniocaudal view; MLO: Mediolateral Oblique view. ^⁎⁎^ROI: Region Of Interest; in this article, it refers to MCC.

The DATA file contains all the information and includes 730 records corresponding to 730 MCCs found in 620 mammograms. Three resolutions are available: 50, 70, and 100 microns. Table 2 presents the total number of mammograms and MCCs for each resolution.

**Table 2:** Summary of mammograms and MCCs by image resolution.

Resolution (mm)	Mammograms	MCCs
0.05	228	234
0.07	358	462
0.1	34	34
Total	620	730

In Table 3, mammograms are grouped by resolution and the number of MCCs they contain. The second column lists mammograms with a single MCC, while the third column shows mammograms with more than one MCC.

**Table 3:** Mammograms grouped by resolution and number of MCCs.

Resolution (mm)	Single MCC	Multiple MCCs	Total
0.05	223	5	228
0.07	358	0	358
0.1	34	0	34
Total	615	5	620

**Fig. 1:**
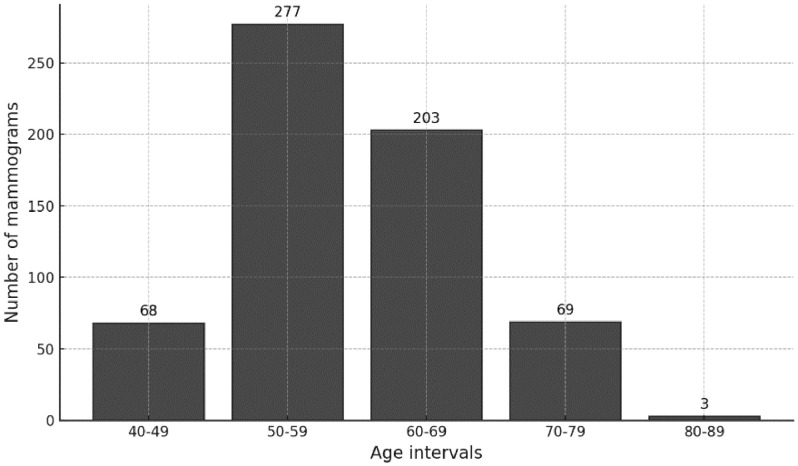
Distribution of mammograms by age intervals.

Table 4 categorizes the patients' ages into intervals: 40–49, 50–59, 60–69, 70–79, and 80–89. Fig. 1 shows the overall distribution for each of these intervals. Note that the representation is based on mammograms rather than individual patients, as one patient may have more than one mammogram.

**Table 4:** Distribution of Mammograms across age intervals and resolutions.

Resolution (mm)	Age intervals					Total
	40 - 49	50 - 59	60 - 69	70 - 79	80 - 89	
0.05	22	84	85	36	1	228
0.07	44	173	109	30	2	358
0.1	2	20	9	3	0	34
Total	68	277	203	69	3	620

Table 5 presents the total number of mammograms with right and left laterality, grouped by resolution.

**Table 5:** Mammograms grouped by resolution and laterality.

Resolution (mm)	Laterality		Total
	Left	Right	
0.05	139	89	228
0.07	183	175	358
0.1	22	12	34
Total	344	276	620

Table 6 presents the total number of mammograms with Craniocaudal (CC) and Mediolateral Oblique (MLO) views, grouped by resolution.

**Table 6:** Mammograms grouped by resolution and view type.

Resolution (mm)	View		Total
	CC	MLO	
0.05	110	118	228
0.07	199	159	358
0.1	17	17	34
Total	326	294	620

## 4 Experimental design, materials and methods

MexBreast was collected at the Radiodiagnóstico e Imagen de Chihuahua Clinic in Chihuahua, Chihuahua, Mexico, through its Picture Archiving and Communication System (PACS). Each imaging device in the clinic is connected to this system, meaning that every time an imaging study is performed, it is automatically stored in the central server in Digital Imaging and Communications in Medicine (DICOM) format, which is the standard for medical imaging. For data storage, the Orthanc DICOM server [2] is used, an open-source solution designed for managing and retrieving medical images. This server contains historical imaging studies from previous years. To access the stored studies, radiologists use OsiriX [3], a specialized medical imaging viewer that serves as the client application to connect with the server. Additionally, radiologists use medical-grade monitors specifically designed for diagnostic imaging to ensure accurate interpretation. Each collected study was interpreted and labeled in a peer-review process by two expert radiologists specializing in breast imaging (Professional License Nos. 10649487, 11204895, issued by the Secretaría de Educación Pública (SEP), Mexico). To be more specific, both radiologists evaluated each mammogram separately, and after their independent assessments, their evaluations were compared. In cases where discrepancies arose, a consensus was reached through discussion to ensure consistency and accuracy in the final labeling. Only studies with lesions identified as MCCs or suspicious microcalcifications classified as BI-RADS 4 [4] were included. The classification of the mammograms with MCCs followed the guidelines of the Breast Imaging Reporting and Data System (BI-RADS) [4], an internationally recognized standard developed by the American College of Radiology (ACR) to ensure consistency in the interpretation and reporting of breast imaging findings. The original files or images were in DICOM format, containing both the images and metadata or interpretations provided by the radiologists. Radiology viewers such as OsiriX allow specialists to annotate images by adding measurements, regions of interest (highlighted with circles or squares), notes, markers, and other annotations, which are stored within the same DICOM file as metadata.

**Fig. 2:**
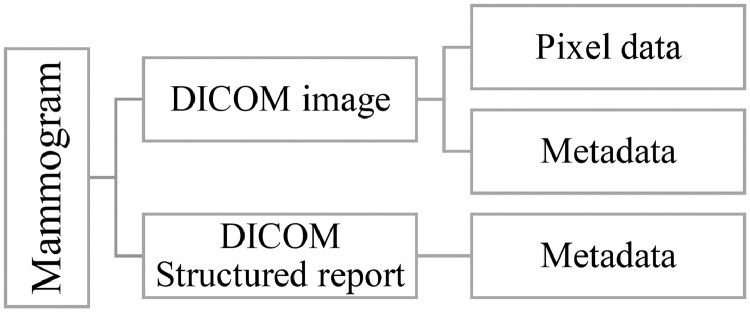
Hierarchical structure of mammogram data based on DICOM source files.

DICOM files use a structured system of tags to store metadata, including patient information, acquisition parameters, and annotations. DICOM Structured Report (SR) files also store structured diagnostic findings, such as lesion descriptions and BI-RADS classifications, ensuring interoperability across medical imaging systems. Fig. 2 illustrates how the information from each collected mammogram is stored.

To convert the Pixel Data of each DICOM image file into PNG format, a Python 3.0 program was developed using the Pydicom library [5]. This library also enabled the extraction of mammogram dimensions, scanner brands, models, and resolutions from the metadata of the DICOM image files [6]. Each DICOM tag has a specific purpose, and its numbering indicates what type of information it represents. This structure is an integral part of the DICOM protocol. In Table 7, we present two columns: Data and Tag. When exploring the metadata of each DICOM file, it consists exclusively of tags and their corresponding values. For example, it is universally established that (0008,0070) corresponds to the Scanner Manufacturer, as shown in the table.

**Table 7:** Tags Used from DICOM image.

Data	Tag (Group, Element)
Scanner Manufacturer	(0008,0070)
Scanner Model	(0008,1090)
Image Height (px)	(0028,0010)
Image Width (px)	(0028,0011)
Pixel Resolution (mm)	(0028,0030)
Patient Age	(0010,1010)
Laterality	(0020,0062)
View	(0018,5101)

The corresponding metadata was extracted and recorded for each DICOM file in the DATA file, consolidating all the relevant information from each mammogram. This extraction process required identifying the correct DICOM tags associated with each attribute in the dataset. Since DICOM metadata consists of predefined tags, it was necessary to know in advance which tags corresponded to each piece of information.

Most of these metadata fields are automatically populated by the scanner at acquisition time. Using the extracted DICOM tags, the DATA file was systematically populated. These attributes and their corresponding DICOM tags are detailed in Table 7.

Additionally, information about the MCCs in each mammogram was retrieved from the DICOM SR files [7] (see Table 8). For each mammogram, the description, BI-RADS code, and lesion coordinates were extracted from the metadata of the DICOM SR files. This information and the data established in Table 7 were consolidated into the DATA file to ensure a comprehensive and structured dataset.

**Table 8:** Tags Used from DICOM Structured report.

Data	Tag (Group, Element)
BI-RADS Code	(0040,A043)
Lesion Description	(0040,A160)
Lesion Coordinates	(0070,0022)
Referenced SOP UID	(0008,1155)

After extracting the images and data, a depuration process was performed. Unlike Scanned Film Mammograms (SFM), which are obtained by digitizing X-ray films and may include artifacts or quality degradation from the scanning process, Full-Field Digital Mammograms (FFDM) capture images directly in digital format, offering higher resolution and improved contrast. In this study, FFDM images were manually selected, focusing on those where the marked lesions covered an area of at least 1 cm², as this corresponds to the size of an MCC [8]. This process was applied to the DATA file by filtering the columns ROI Height (H) and ROI Width (W) based on the Resolution (R) of the mammograms. Note that the displayed DATA file represents the curated version with filtered information. Equation 1 illustrates this filtering process:

Equation 1. Filtering criteria for ROI dimensions based on mammogram resolution.

$$H\geq \frac{10}{R}\;\text{and}\;W\geq \;\frac{10}{R}$$

where:

• H and W are the height and width of the ROI (MCC) in pixels.
• R is the resolution in mm/pixel.

For instance:

• At 50 microns (R = 0.05): H ≥ 200 and W ≥ 200
• At 70 microns (R = 0.07): H ≥ 143 and W ≥ 143
• At 100 microns (R = 0.1): H ≥ 100 and W ≥ 100

**Fig. 3:**
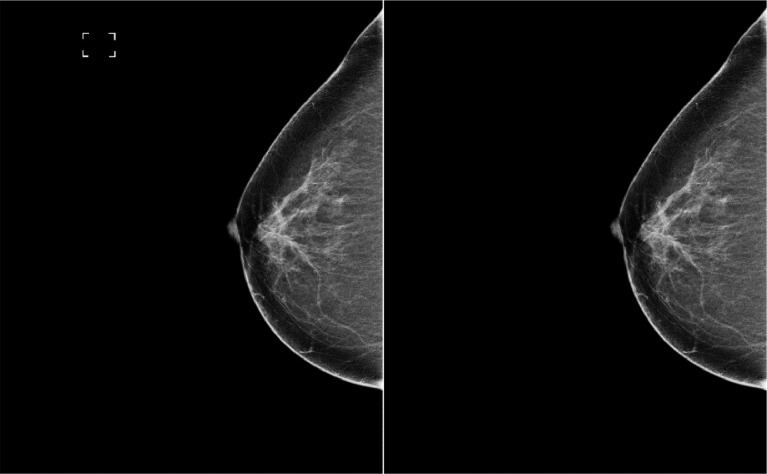
Comparison of mammogram IMG_00231 with and without a printed label (left: with a label, right: without a label).

A significant portion of the mammograms contained printed labels, such as empty brackets, mammogram laterality, vertical lines, and other elements that could introduce noise or require additional preprocessing for removal. The labels were manually removed to prevent this and assist researchers using this database. GIMP [9], a graphics editing program was used to open each mammogram, where the printed labels were identified and manually erased. It is important to note that all labels were located outside the breast region, ensuring no modifications were made to the breast tissue content. 71ted tasks were employed to ensure that the content of the mammograms was not altered or removed in any way. Fig. 3 shows an example of a mammogram with and without the printed label.

**Fig. 4:**
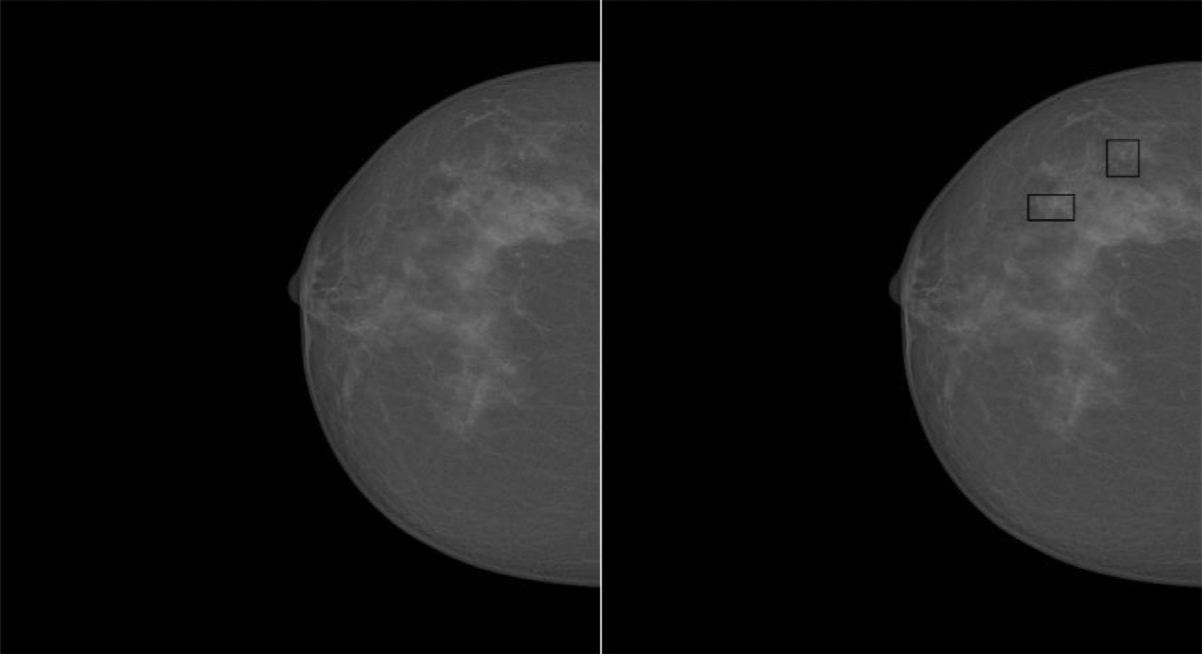
Comparison of mammogram IMG_00001 without (left) and with (right) marked MCCs.

In addition to the DATA file, which contains all the necessary information for working with mammograms, and the UNMARKED MAMMOGRAMS folder, which includes all mammograms with MCCs, we decided to include the MARKED MAMMOGRAMS folder. This folder contains the same mammograms as those in the UNMARKED MAMMOGRAMS folder but with the MCCs highlighted using black boxes. This addition was made to provide researchers with an easy reference for locating the lesions without requiring additional programming. This task was performed using the OpenCV library [10] with the drawContours method, where the input data consisted of the lesion coordinates included in the DATA file. Fig. 4 shows an example of a mammogram without MCC identification stored in the UNMARKED MAMMOGRAMS folder and the same mammogram with MCC identification stored in the MARKED MAMMOGRAMS folder.

Table 9 shows publicly available mammography databases containing MCCs. It can be observed that MEXBreast is the most extensive dataset in terms of the number of mammograms with MCCs among those that exclusively contain FFDM. Additionally, it is the only dataset that includes three different resolutions and does not provide printed labels.

**Table 9:** Publicly available mammography databases containing MCCs.

Database	Mammograms with MCCs	Resolution (mm)	Image Type	Printed Labels
[11] **CBIS-DDSM**	738	Not specified	SFM	Y
[12] **INbreast**	21	0.07	FFDM	N
[13] **BCDR**	Not specified	Not specified	SFM	Y
[14] **MIAS**	13	0.2	SFM	Y
**MEXBreast**	620	0.05 (228), 0.07 (358), 0.1 (34)	FFDM	N

As a baseline, we evaluated our previously developed CNN model [1], which was trained on the INbreast dataset at a resolution of 70 microns, achieving an accuracy of 99.74%. When tested on MEXBreast mammograms with the same resolution, the model obtained a similar accuracy of 99.8%.

## Limitations

• The only lesion types considered in this dataset are MCCs. Whereas BI-RADS category 4 includes a range of suspicious lesions, here it refers exclusively to MCCs.
• All mammograms were acquired exclusively using scanners at the Radiodiagnóstico e Imagen de Chihuahua Clinic, following the acquisition protocols specific to that institution. No additional sources or data distributions are included in the dataset.
• Although the dataset includes mammograms at three resolutions, the number of cases available at 100-micron resolution is limited, which may lead users to apply data augmentation techniques when training deep learning models.

## Ethics statement

This research involved human subjects, and informed consent was obtained from all participants. Conducted in accordance with the principles outlined in the Declaration of Helsinki, the research received approval from the Ethics Committee of the Radiodiagnóstico e Imagen de Chihuahua Clinic in Chihuahua, Chihuahua, Mexico, under protocol number CE-2024-001, ensuring compliance with all relevant ethical and legal guidelines. All patient information was handled anonymously, except for age, which does not constitute any form of patient identification. Additionally, it was ensured that each image contained no embedded labels or identifying information to maintain the highest level of confidentiality, and the authors confirmed adherence to all ethical requirements and guidelines for scientific publication.

The dataset is released under the Creative Commons Attribution 4.0 International (CC BY 4.0) license, which allows sharing, modification, and commercial use, provided proper credit is given, a link to the license is included, and any modifications are indicated.

## Credit author statement

**Ricardo Salvador Luna Lozoya:** Conceptualization, Methodology, Software, Validation, Formal analysis, Investigation, Resources, Data Curation, Writing - Original Draft, Visualization, Project administration. **Karina Núñez Barragán:** Conceptualization, Methodology, Validation, Formal analysis, Investigation, Resources, Data Curation, Writing - Original Draft, Supervision. **Humberto de Jesús Ochoa Domínguez:** Conceptualization, Methodology, Validation, Formal analysis, Investigation, Resources, Writing - Original Draft, Visualization, Project administration, Writing - Review & Editing, Supervision, Funding acquisition. **Juan Humberto Sossa Azuela:** Writing - Original Draft, Writing - Review & Editing, Supervision, Validation. **Vianey Guadalupe Cruz Sánchez:** Writing - Original Draft, Writing - Review & Editing, Supervision, Validation. **Osslan Osiris Vergara Villegas:** Writing - Original Draft, Writing - Review & Editing, Supervision, Validation.

## Data Availability

Mendeley DataMexican dataset of digital mammograms (MEXBreast) with suspicious clusters of microcalcifications (Original data)
